# The CanPain SCI clinical practice guidelines for rehabilitation management of neuropathic pain after spinal cord injury: 2021 update

**DOI:** 10.1038/s41393-021-00744-z

**Published:** 2022-02-05

**Authors:** Eldon Loh, Magdalena Mirkowski, Alexandria Roa Agudelo, David J. Allison, Brooke Benton, Thomas N. Bryce, Sara Guilcher, Tara Jeji, Anna Kras-Dupuis, Denise Kreutzwiser, Oda Lanizi, Gary Lee-Tai-Fuy, James W. Middleton, Dwight E. Moulin, Colleen O’Connell, Steve Orenczuk, Patrick Potter, Christine Short, Robert Teasell, Andrea Townson, Eva Widerström-Noga, Dalton L. Wolfe, Nancy Xia, Swati Mehta

**Affiliations:** 1 Lawson Health Research Institute, London, ON Canada; 2 Parkwood Institute, London, ON Canada; 3 Western University, London, ON Canada; 4 St Joseph’s Health Care Pain Management Program, London, ON Canada; 5 Icahn School of Medicine at Mount Sinai, New York City, NY USA; 6 University of Toronto, Toronto, ON Canada; 7 Ontario Neurotrauma Foundation, Toronto, ON Canada; 8 SCI-Ontario, Toronto, ON Canada; 9 John Walsh Centre for Rehabilitation Research, Sydney, NSW Australia; 10 The University of Sydney, Sydney, NSW Australia; 11 Stan Cassidy Centre for Rehabilitation, Fredericton, NB Canada; 12 Dalhousie University, Halifax, NS Canada; 13 Nova Scotia Health Authority, Halifax, NS Canada; 14 University of British Columbia, Vancouver, BC Canada; 15 The Miami Project, Miami, FL USA; 16 University of Miami Miller School of Medicine, Miami, FL USA

**Keywords:** Health policy, Neuropathic pain

## Abstract

**Study design:**

Clinical practice guidelines.

**Objectives:**

The objective was to update the 2016 version of the Canadian clinical practice guidelines for the management of neuropathic pain in people with spinal cord injury (SCI).

**Setting:**

The guidelines are relevant for inpatient, outpatient and community SCI rehabilitation settings in Canada.

**Methods:**

The guidelines were updated in accordance with the Appraisal of Guidelines for Research and Evaluation II tool. A Steering Committee and Working Group reviewed the relevant evidence on neuropathic pain management (encompassing screening and diagnosis, treatment and models of care) after SCI. The quality of evidence was scored using Grading of Recommendations Assessment, Development and Evaluation (GRADE). A consensus process was followed to achieve agreement on recommendations and clinical considerations.

**Results:**

The working group identified and reviewed 46 additional relevant articles published since the last version of the guidelines. The panel agreed on 3 new screening and diagnosis recommendations and 8 new treatment recommendations. Two key changes to these treatment recommendations included the introduction of general treatment principles and a new treatment recommendation classification system. No new recommendations to model of care were made.

**Conclusions:**

The CanPainSCI recommendations for the management of neuropathic pain after SCI should be used to inform practice.

## Introduction

Neuropathic pain (NP) presents significant challenges to those living with spinal cord injury (SCI), with negative effects on function participation, (e.g., mood, sleep), and quality of life. For clinicians, providing support for those with NP after SCI continues to be a challenge. The 2016 CanPainSCI Clinical Practice Guidelines (CPG) formalized a series of recommendations for the rehabilitation management of NP after SCI [1]. These guidelines were developed in accordance with the Appraisal of Guidelines for Research & Evaluation (AGREE) II tool [2], and focused on three domains: screening and diagnosis [3], treatment (both pharmacologic and non-pharmacologic) [4], and models of care [5].

The current document presents updates to the CanPainSCI CPG based on additional relevant literature that has been published since the first iteration of the guidelines. The ongoing goals of these CPG are to (1) provide practical and actionable guidelines with a strong rating on the AGREE-II scale, (2) support standardized care in the rehabilitation management of NP after SCI, and (3) identify opportunities for further research in this area.

As in the 2016 CPG, an international group of experts formed a Working Group (WG) that would update the CanPainSCI guidelines under the direction of the Steering Committee (SC).

## Scope and purpose

The scope and purpose of these guidelines are unchanged from the 2016 version. The target population to which these CPG apply includes adults living with SCI who are in the subacute phase of inpatient rehabilitation management to those living in the community. It excludes those being cared for in an acute care setting immediately after their initial injury. These guidelines are intended to be used by clinicians who care for those with SCI. Researchers and health policy experts will also find these guidelines valuable.

## Methods

The overall CPG update process was overseen by the SC (EL, ARA, MM, JWM, SM) with ongoing input and review from the WG. A facilitator (EJM) assisted with organizing and planning meetings. The CPG update methodology followed a similar process as the initial 2016 CPG, in accordance with the AGREE II tool. The main steps of this process involved: (1) identifying experts for the new 2020 WG, (2) updating literature search and review, (3) evaluating the quality of evidence using a modified Grading of Recommendations Assessment, Development and Evaluation (GRADE) approach, (4) discussing the updated evidence with WG members to develop and suggest modifications to existing recommendations or to propose new recommendations, and (5) voting on the recommendations and guidelines. The update was funded by the Ontario Neurotrauma Foundation (ONF); guideline development was editorially independent from the funder.

### Working group composition

Experts who were involved in the initial 2016 CanPainSCI CPG WG were invited to participate in the 2020 WG. New members were recruited if previous WG members were unable to participate or if their discipline/area of professional expertise was not represented in the 2016 WG composition. Representation from a broad spectrum of disciplines that reflected a national and international geographic distribution was sought. Experts from physiatry, pain medicine, psychology, pharmacy, occupational therapy, physical therapy, nursing, and research were included. Those with lived experience were also included in the WG, as were representatives from the ONF and a peer advocacy organization (SCI-Ontario) (Table 1). WG members were asked to provide a list of potential conflicts of interest prior to participating in meetings. If a member was felt to have a potential conflict with an item to be voted on or discussed, they could voluntarily withdraw from voting/discussing that item. If the chair assessed that a member may have a conflict of interest on a particular item, the chair would ask a particular member to withdraw/abstain from discussing that specific item. Prior to final submission of the manuscript, WG members again submitted forms identifying potential conflicts of interest (Table 2).

**Table 1:** CanPainSCI Working Group.

Member	Affiliation	Professional role
ARA^a^	Lawson Health Research Institute, London, ON, Canada	Research Assistant
BB RDH, BSc^a^	Icahn School of Medicine at Mount Sinai, New York City, NY, USA	Clinical Research Coordinator
TNB MD	Department of Rehabilitation and Human Performance, Icahn School of Medicine at Mount Sinai, New York City, NY, USA	Professor, Physiatrist
SG, PT, PhD	University of Toronto, Toronto, ON, Canada	Physiotherapist, Assistant Professor
TJ, MD	Ontario Neurotrauma Foundation, Toronto, On, Canada	Program Director, Lived Experience
AK-D RN, MScN, CNNC,CRN	Parkwood Institute, London, ON, Canada	Clinical Nurse Specialist
DK, BScPhm, PharmD, ACPR	St. Joseph’s Health Care Pain Management Program, London, ON, Canada	Clinical pharmacist
OL	SCI-Ontario, Toronto, ON, Canada	Lived Experience
GL-T-F	Parkwood Institute, London, ON, Canada	Occupational Therapist
EL MD, FRCP(C)^b^	Lawson Health Research Institute, St. Joseph’s Health Care Pain Management Program, Western University, London, ON, Canada	Physiatrist, Associate Professor
SM MA, PhD^a^	Lawson Health Research Institute, Western University, London, ON, Canada	Psychologist, Scientist
MM MSc, MScOT, OT Reg. (Ont.)^a^	Parkwood Institute Research, Lawson Health Research Institute, London, ON, Canada	Research Fellow
DEM MD, FRCP(C)	St. Joseph’s Health Care Pain Management Program, Western University, London, ON, Canada	Professor, Earl Russell Chair, Pain Research, Neurologist
CO’C MD, FRCP(C)	Stan Cassidy Centre for Rehabilitation and Dalhousie University Faculty of Medicine, Fredericton, NB, Canada	Physiatrist, Research Chief, Assistant Professor
SO PsyD	Parkwood Institute, London, ON, Canada	Psychologist
PP MD, FRCP(C)	Western University, Parkwood Institute, London, ON, Canada	Physiatrist
JWM MBBS, PhD, FAFRM(RACP)	John Walsh Centre for Rehabilitation Research, The University of Sydney, Sydney, NSW, Australia	Professor, Physiatrist
CS MD, FRCP(C), FACP	Dalhousie University and Nova Scotia Health Authority, Halifax, NS, Canada	Department head/ Chief Medicine, Associate Professor, Physiatrist
RT MD, FRCP(C)	Lawson Health Research Institute, Western University, London, ON, Canada	Physiatrist, Scientist
AT MD, FRCPC, MScHPEd	University of British Columbia, Vancouver, BC, Canada	Clinical Professor, Physiatrist
EW-N DDS, PhD	The Miami Project, University of Miami, Miller School of Medicine, Miami, FL, USA	Research Professor
DLW PhD	Parkwood Institute Research, Lawson Health Research Institute, London, ON, Canada	Scientist
NX	SCI-Ontario, Toronto, ON, Canada	Lived Experience
DJA^a^	Lawson Health Research Institute, London, ON, Canada	Research Associate

^a^Steering committee.

^b^Chair.

**Table 2:** Disclosures of the CanPainSCI CPG group.

Member	Disclosures
ARA	ARA has nothing to disclose.
DJA	DJA has nothing to disclose.
BB	BB has nothing to disclose
TNB	TNB has nothing to disclose.
SG	SG has nothing to disclose.
TJ	TJ is employed by the Ontario Neurotrauma Foundation.
AK-D	AK-D has nothing to disclose.
DK	DK has nothing to disclose.
OL	OL has nothing to disclose.
GL-T-F	GL-T-F has nothing to disclose.
EL	EL has nothing to disclose.
SM	SM has nothing to disclose.
MM	MM has nothing to disclose.
DEM	DEM reports personal fees from Canopy Growth Inc, outside the submitted work.
CO’C	CO’C reports grants from Praxis Spinal Cord Institute, other from Cytokinetics, other from Orion, other from Mallinckrodt, grants from New Brunswick Health Research Foundation, personal fees from Spectrum/Canopy, personal fees from Shoppers Drug Mart, grants and personal fees from IPSEN, personal fees from MT Pharma, personal fees from Tilray, personal fees from Allergan, personal fees from Roche, outside the submitted work.
SO	SO has nothing to disclose.
PP	PP has nothing to disclose.
JWM	JWM has nothing to disclose.
CS MD	CS has nothing to disclose.
RT	RT reports other funding from Allergan (Predictive Model for Treatment of Spasiticity Post Stroke (Botox), chair positions on data monitoring/advisory boards for studies on statins for osteoporosis after SCI and exercise in SCI, and medicolegal work for assessment of individuals with whiplash and other musculoskeletal injuries after motor vehicle accidents outside the submitted work.
AT	AT has nothing to disclose.
EW-N	EW-N has nothing to disclose.
DLW	DLW has nothing to disclose.
NX	NX has nothing to disclose.

### Updated literature search

A comprehensive literature search update was conducted for articles published from November 1, 2013 to October 30, 2018, using the following scientific databases: MEDLINE, EMBASE, CINAHL, PsycInfo, and Cochrane Library (Central Register of Controlled Trials). ‘Spinal cord injury’ and ‘neuropathic pain’ key words were searched in combination with ‘intervention’, ‘diagnosis’, and ‘model of care’ key words (see Appendix 1). Medical Subject Headings were used as available in each database. Searches were limited to articles published in the English language.

#### Inclusion/Exclusion criteria

Articles which investigated interventions for the treatment of NP in people with SCI were included if they met the following inclusion criteria: (1) the study population was comprised of ≥50% individuals with traumatic or non-traumatic SCI, (2) the study population had NP or mixed pain, (3) there were ≥3 human adult participants (≥18 years) with SCI and neuropathic or mixed pain, (4) the study was conducted in a rehabilitation, outpatient, or community setting, (5) the effect of treatment on pain intensity was assessed. Articles which investigated screening or diagnostic tools or models of care for NP in people with SCI were also included. Articles were excluded if: (1) they involved participants with musculoskeletal pain only, (2) were conducted in an acute setting, (3) were reviews, case studies/reports, study protocols, or qualitative studies.

#### Article selection

After removing duplicates, articles were first screened for eligibility based on title and abstract, then based on full text, by two independent reviewers (MM, BB). Any discrepancies were resolved by a third reviewer (EL). Additionally, any other articles identified following the review of the reference lists of articles identified using the search strategy or those which were deemed to be of relevance by the expert panel were included.

#### GRADE assessment

The methodological quality of each article was assessed by two independent reviewers (MM, BB, EL, or SM) using a modified GRADE approach. Pain intensity was the primary outcome assessed with GRADE for articles investigating treatment modalities. If agreement on an individual GRADE level assignment was not achieved, discrepancies were resolved by a third reviewer. WG members provided input on the assessment of evidence quality during their discussions. If a change in the assigned quality of evidence was proposed, the WG voted on a potential change. If 75% or more of the panellists agreed, the GRADE assignment was adjusted.

GRADE criteria were modified in an identical process as the original 2016 CPG. Due to the generally smaller sample size in the SCI literature, the reviewers did not necessarily downgrade the quality of evidence for studies with a small sample size (see Appendix 2 GRADE scoring criteria) [6].

### Working group discussions

The overall WG was divided into four smaller discussion groups that met two to four times via an online videoconferencing platform (Zoom Technologies Inc.™) from January 2020 to April 2020. Each group was assigned different treatments for which new evidence was available. One group was assigned additional literature to review related to diagnosis and screening. Each group was provided with evidence tables that summarized existing literature for that treatment (if any), updated and new literature, and the modified GRADE rating. Groups reviewed the new and existing literature and provided input on the modified GRADE assignment. If there was disagreement over a particular treatment’s evidence base because of conflicting study results, the WG could request a meta-analysis to attempt to resolve this conflict. New and modified recommendations were proposed by the small discussion groups for presentation to the full WG.

In accordance with the GRADE process and the AGREE II tool, the groups assigned a GRADE rating and a level of strength (strong or weak) to each proposed recommendation. Clinical experience, side-effect profile, effectiveness in other populations with NP, and any other factors that the panel considered relevant were used to determine the strength of each recommendation.

Proposed recommendations were summarized and presented to the WG for discussion at two additional meetings attended by all members. These meetings (2–3 h) were held over videoconference (Zoom Technologies, Inc.™) on June 15 and June 25, 2020. The WG was divided into small groups. Each group reviewed and discussed all new and modified recommendations. Groups also reviewed all 2016 treatment recommendations for which there was new evidence but no new or modified recommendations, to ensure that this was appropriate. The WG also discussed the best means with which to present and frame the recommendations. A full group discussion was held at the end of each meeting to summarize each group’s key discussion points.

Proposed new and modified recommendations arising from the final meetings were summarized for presentation to the panel. The WG anonymously voted on the format of the CPG, any new/modified recommendations, GRADE ratings of the recommendations (if disagreement was voiced during the WG discussions) and the strength of new/modified recommendations using online survey software (SurveyMonkey^®^). As for the 2016 CPG, recommendations achieving at least 75% agreement were adopted. Remaining treatments with new evidence were designated as “requiring further research” if appropriate and are summarized within the CPG. Existing recommendations from the 2016 CPG for which there was no new evidence remain unchanged in the current edition.

#### External review and endorsement

The complete drafted supplement was sent for external review in January of 2021. The quality of the guidelines was assessed by 5 external reviewers (Table 3); each of whom approved the guidelines and provided feedback. Further, 4 of 5 reviewers completed the Agree II tool and provided scores on quality (Table 4). The manuscript was revised based on suggestions from these reviewers. The 2021 update to the CanPainSCI guidelines are endorsed by SCI Canada, the Canadian Association of Physical Medicine and Rehabilitation (CAPMR) SCI Special Interest Group (SIG), the CAPMR Pain SIG, the Canadian Spinal Research Organization, Spinal Cord Injury Ontario (SCI-Ontario), and Ability New Brunswick (Ability NB).

**Table 3:** External reviewers of the CanPainSCI 2021 Clinical Practice Guideline Update.

Member	Affiliation	Professional role
Andréane Richard-Denis, MD, MSc, FRCPC	Université de Montréal, Montreal QC, Canada	Physiatrist and scientist
Brenda Lau, MD, FRCPC, FRCPC (Pain) founder, MM, CIPS	Vancouver BC, Canada	Anesthetist, Pain Medicine
Neal McKinnon, MSc (PT), PhD	Parkwood Institute, London ON, Canada	Physiotherapist
James Milligan, MD, CCFP	Mobility Clinic, Waterloo ON, Canada	Family Medicine
Keith Sequeira, MD, FRCPC	Western University, London ON, Canada	Physiatrist

**Table 4:** Agree II Tool Scores.

Scope and purpose	Mean score
Item 1	7.0
Item 2	6.0
Item 3	7.0
**Stakeholder involvement**	
Item 4	6.5
Item 5	6.3
Item 6	6.5
**Rigor of development**	
Item 7	6.8
Item 8	6.8
Item 9	5.8
Item 10	7.0
Item 11	6.5
Item 12	6.8
Item 13	6.0
Item 14	7.0
**Clarity of presentation**	
Item 15	7.0
Item 16	6.3
Item 17	7.0
**Applicability**	
Item 18	6.5
Item 19	6.3
Item 20	6.0
Item 21	6.3
**Editorial independence**	
Item 22	7.0
Item 23	7.0
**Overall**	
Overall quality	6.3
Recommend guideline for use?	Yes

## Results

For the 2021 CanPainSCI CPG, the panel reviewed 46 additional articles published since the last version of the guidelines which met inclusion criteria or were deemed to be of relevance by the panel. The panel agreed on three new screening and diagnosis recommendations, and eight new treatment recommendations. No new model of care recommendations were proposed as no relevant articles were identified.

Within the treatment recommendations, seven of the 10 existing recommendations from 2016 were re-evaluated because new relevant studies published; however, no changes were made to these recommendations. Additionally, 12 new treatment options not evaluated in 2016 were assessed by the CanPainSCI group but were found not to warrant a recommendation at this time. Five treatment modalities evaluated in 2016 that did not warrant a recommendation at the time, but for which new evidence was available, were also reviewed in this version; however, no recommendations were made for these treatments.

For the screening and diagnosis recommendations, only the three new recommendations are discussed in this document. Detailed discussion on the other recommendations can be found in the 2016 version of the CanPainSCI CPG (https://www.nature.com/articles/sc201689). Given the model of care recommendations have not changed, please refer to the 2016 version (https://www.nature.com/articles/sc201691). As there has been extensive reframing of the treatment recommendations for the 2021 update, the entire treatment guidelines are presented in this document, including recommendations and treatments requiring further research that are unchanged from 2016. Key 2021 updates are identified within the guideline text.

This update replaces the 2016 recommendations for treatment [4] and serves to supplement the 2016 screening and diagnosis recommendations [3] and 2016 recommendations for model systems of care [5]. Table 5 provides an updated list of all CanPainSCI recommendations.

**Table 5:** Summary of 2021 CanPainSCI Recommendations^a^.

#	Screening and Diagnosis Recommendations	Quality of evidence	Strength of Recommendation
1.1	All patients with spinal cord injury must be screened for pain using a simple yes/no question.	Expert opinion	N/A
1.2	Any member of the health-care team can, and should, screen for the presence of pain.	Expert opinion	N/A
1.3	Screening for pain should occur on admission to rehabilitation, regularly during inpatient rehabilitation and after discharge at each follow-up.	Expert opinion	N/A
1.4	If pain is present at screening, an assessment to determine the type of pain, its intensity and interference should be carried out.	Expert opinion	N/A
1.5	Diagnosis of neuropathic pain, including its causes, should be informed by (1) a complete patient history, (2) a physical examination, (3) the International Spinal Cord Injury Pain (ISCIP) Classification system, and (4) investigations.	Expert opinion	N/A
*1.6*	*The SCIPI and NPSI can be used to supplement the diagnosis of neuropathic pain for people with spinal cord injury.*	*High*	*Strong*
1.7	Assess for serious underlying conditions (red flags) that may cause, aggravate, or mimic neuropathic pain and that require further investigation and prompt medical review.	Expert opinion	N/A
1.8	Assess and manage psychosocial factors (yellow flags) that may contribute to pain-related distress and disability.	Expert opinion	N/A
1.9	The International Spinal Cord Injury Pain Basic Data Set (ISCIPBDS) v2.0 should be used as a standardized tool for assessing and documenting pain in patients with spinal cord injury.	Expert opinion	N/A
*1.10*	*The NPSI can be used to supplement the assessment and documentation of neuropathic pain.*	*High*	*Strong*
1.11	Address patient concerns, expectations and needs as part of the neuropathic pain assessment.	Expert opinion	N/A
1.12	Standardized evaluation of treatment response should be carried out by the health-care team at regular intervals.	Expert opinion	N/A
1.13	The evaluation of treatment response should include assessment of changes in pain intensity, mood and function using the International Spinal Cord Injury Pain Basic Data Set v2.0. Evaluation also includes assessment of adverse events, aberrant behavior and compliance.	Expert opinion	N/A
1.14	All patients with new-onset or worsening pain need to be reassessed.	Expert opinion	N/A
*1.15*	*The NPSI can be used to supplement the evaluation of treatment response.*	*High*	*Strong*

**#**	**Treatment Recommendations**	**Quality of Evidence**	**Strength of Recommendation**
General Treatment Principles			
*G1*	*Those with NP after SCI should be encouraged to pursue self-management strategies that they find beneficial for pain intensity reduction, coping with pain, and improving functional abilities.*	*Expert Opinion*	*N/A*
*G2*	*A comprehensive pain management strategy should address issues with activity, sleep, and mood that result from, or may worsen, pain. This could include both pharmacologic and non-pharmacologic strategies, as appropriate.*	*Expert Opinion*	*N/A*
*G3*	*Consider referral for specialized multidisciplinary SCI rehabilitation management to address functional limitations including activity, mood, and sleep.*	*Expert Opinion*	*N/A*
*G4*	*An interdisciplinary pain program that may consist of patient education, cognitive behavioural therapy, self-management strategies, group discussions, exercise, and other modalities could be considered in those with spinal cord injury and neuropathic pain.*	*Expert Opinion*	*N/A*
*G5*	*Cognitive behavioural therapy could be considered to improve coping skills and reduce pain interference.*	*Expert Opinion*	*N/A*
First Line Treatments			
2.1	Pregabalin should be used for the reduction of neuropathic pain intensity among persons with spinal cord injury.	High	Strong
2.2	Gabapentin should be used for the reduction of neuropathic pain intensity among persons with spinal cord injury.	High	Strong
2.3	Amitriptyline can be used for the reduction of neuropathic pain intensity among persons with spinal cord injury.	High	Strong
B Options			
*2.4*	*Oxcarbazepine can be used for the reduction of neuropathic pain intensity after spinal cord injury.*	*High*	*Strong*
2.5	Tramadol can be used for the reduction of neuropathic pain intensity among persons with spinal cord injury.	Moderate	Strong
2.6	Lamotrigine can be considered in those with incomplete spinal cord injury for the reduction of neuropathic pain intensity.	Moderate	Strong
C Options			
*2.7*	*Botulinum toxin A may be considered in the management of below level neuropathic pain after SCI, with injection localized to the area of maximal pain.*	*High*	*Weak*
2.8	Transcranial direct current stimulation (tDCS) may be considered for reducing neuropathic pain intensity among persons with spinal cord injury.	High	Weak
2.9	Combined visual illusion and transcranial direct current stimulation may be considered for reducing neuropathic pain intensity among persons with spinal cord injury.	High	Weak
D Options			
*2.10*	*Cannabinoids may be considered for the management of neuropathic pain among persons with spinal cord injury.*	*Moderate*	*Weak*
2.11	Transcutaneous electrical nerve stimulation (TENS) may be considered for the reduction of neuropathic pain intensity among persons with spinal cord injury.	Low	Weak
2.12	Oxycodone may be considered for the reduction of neuropathic pain intensity among persons with spinal cord injury.	Moderate	Weak
2.13	The dorsal root entry zone (DREZ) procedure may be considered in exceptional circumstances and as a last resort for reducing neuropathic pain intensity among persons with spinal cord injury.	Low	Weak
Advise Against Use			
2.14	Levetiracetam should not be used for reducing neuropathic pain intensity among persons with spinal cord injury.	High	Strong
2.15	Mexiletine should not be used for reducing neuropathic pain intensity among persons with spinal cord injury.	High	Strong

**#**	**Model Systems of Care Recommendations**	**Quality of evidence**	**Strength of Recommendation**
3.1	Delivery of care for neuropathic pain in people with spinal cord injury should be (1) coordinated, (2) interprofessional, (3) timely, (4) patient centered, (5) using a biopsychosocial framework and (6) evidence based.	Expert opinion	N/A
3.2	A person with spinal cord injury and either (1) new onset or worsening spinal cord injury-related neuropathic pain, and/or (2) ongoing pain that is difficult to manage and/or (3) dissatisfaction with their current pain management protocol should be screened and assessed by a clinician with experience in managing people with spinal cord injury.	Expert opinion	N/A
3.3	Multidisciplinary care coordinated through a spinal cord injury rehabilitation team is recommended when significant functional impacts and/or significant psychological comorbidity factors resulting from neuropathic pain need to be addressed. Further, a detailed plan of care shared among health-care providers needs to be implemented across primary, secondary and tertiary services.	Expert opinion	N/A
3.4	A person with neuropathic pain as a result of spinal cord injury should be discharged from specialized care when three conditions are met: (1) a stable plateau has been reached in pain severity and/or pain-related functional status; (2) an ongoing plan linked to resources and provider follow-up is in place; and (3) self-management techniques have been taught.	Expert opinion	N/A
3.5	The spinal cord injury rehabilitation team should engage in continuous quality improvement, including evaluation and feedback efforts regarding their pain management practices based on patient outcomes.	Expert opinion	N/A

*SCIPI* Spinal Cord Injury Pain Instrument, *NPSI* Neuropathic Pain Symptom Inventory.

^a^New recommendations are in italic.

### Screening and diagnosis recommendations

For screening and diagnosis, three new recommendations were developed as part of the 2021 CanPainSCI CPG update to supplement existing 2016 recommendations (Table 5). The existing recommendations remain “expert opinion” as in the 2016 CPG.

#### Listing of 2021 new screening and diagnosis recommendations

1.6. The Spinal Cord Injury Pain Instrument (SCPI)) and Neuropathic Pain Symptom Pain Inventory (NPSI) can be used to supplement the diagnosis of neuropathic pain for people with spinal cord injury.

1.10 The NPSI can be used to supplement the assessment and documentation of neuropathic pain.

1.15 The NPSI can be used to supplement the evaluation of treatment response.

Level of Evidence: High

Strength of Recommendation: Strong

#### 2021 clinical considerations

Three new studies were identified that addressed screening and diagnosis of NP after SCI [7–9]. Two cross-sectional studies of moderate quality evaluated the SCIPI. One study compared the SCIPI to a clinical assessment of NP [8], with sensitivity of 78%, specificity of 73%, and diagnostic accuracy of 76% when at least two out of four SCIPI items highly correlated with NP were endorsed (electrical/shock like; pins/needles, tingling; skin feels hot/burning, cold?; occurs in insensate areas). A second study evaluated a German language version of SCIPI and the painDETECT questionnaire using the International Association for the Study of Pain (IASP) grading system, with strong convergent construct validity [7].

A 2020 study [9] of the NPSI was proposed by members of the WG for evaluation. Although this 2020 study fell outside the formal literature search window identified in the current CPG, the WG agreed that it was important to consider. There were very few studies on these types of tools in the SCI literature and the WG felt that it may provide important context and guidance for NP screening and diagnosis. This study evaluated the NPSI against a numeric rating scale for pain severity, and the Multidimensional Pain Inventory pain severity and perceived support subscales in those with moderate to severe NP after SCI, with the NPSI demonstrating good psychometric properties in this population. A test-retest reliability (intraclass correlations) of 0.65–0.73 for NPSI subscores and 0.79 for total NPSI; internal consistency with Cronbach’s alpha of 0.70; and construct validity with positive correlations between pain intensity NRS and the pain severity subscale of the MPI (*r* > 0.4) were described [9].

As all three studies are preliminary with relatively small sample sizes, they are not sufficient to change or replace the current tools recommended for screening, making a diagnosis, and evaluating treatment response in NP after SCI. However, they provide additional tools and criteria to consider within these domains.

## Treatment recommendations

### 2021 key changes

#### General treatment principles and treatment recommendation classification

The WG agreed that it was important to formalize key treatment principles within the treatment recommendations to inform clinical practice. Some of these principles were presented in the 2016 version of the CPG, although were not specific recommendations, in contrast to the 2021 version.

In order to assist CPG users and clinicians managing NP after SCI, two key changes were introduced to the treatment recommendations: the addition of five recommendations that are “general treatment principles”, and a major restructuring of the classification of treatment options.

The consensus panel discussed the optimal way to present the recommendations to support and facilitate with implementation of the CanPainSCI CPG into clinical practice. Clearer definitions and guidance regarding the use of different lines of treatment (i.e. first, second, third, and fourth) were found to be necessary.

The panel agreed that the first-line treatments pregabalin and gabapentin should always be considered initially. While amitriptyline is also first-line, its use needs to be carefully considered given the potential side effects of the medication (see section on first-line treatments).

For second-, third- and fourth-line treatments, the panel agreed a hierarchical approach to these treatments was not necessarily appropriate as the overall body of evidence for non-first-line treatments is limited. The panel did not feel it was necessary or appropriate to have exhausted second-line treatments; for example, before initiating a third-line treatment. Clinician selection between second to fourth line treatments could be based on other relevant factors, including patient preference and shared decision-making, tolerability, clinician experience, side effect profile, and accessibility. One exception is the dorsal root entry zone procedure (DREZ), which given its invasiveness as a treatment and significant risk of side effects, should only be considered as a last resort if it is used at all.

The panel emphasized it was, however, still important to acknowledge that certain treatment options had a higher evidence basis and/or strength of recommendation from the expert panel than others (e.g., second-line vs. third- or fourth-line treatments). Thus, a distinction should still be present within the CPG between different groups of treatment options. Given that a hierarchical approach was not used for these treatment groupings, second- to fourth-line treatments were reclassified as “B options” (formerly second-line), “C options” (formerly third-line) and “D options” (formerly fourth-line). The criteria for classifying a treatment within one of these groupings were the same as for second/third/fourth-line treatments, and were based on GRADE/strength of the recommendation as follows (GRADE; strength of recommendation): B options (High/Moderate; Strong); C options (High/Moderate; Weak); D options (Moderate/Low; Weak). The phrasing of recommendations was also standardized to reflect the assessments of the expert panel on the different treatment groupings. B option recommendations are phrased as “can be used” for NP following SCI, while C and D option recommendations are phrased as “may be considered” for NP following SCI.

**Fig. 1:**
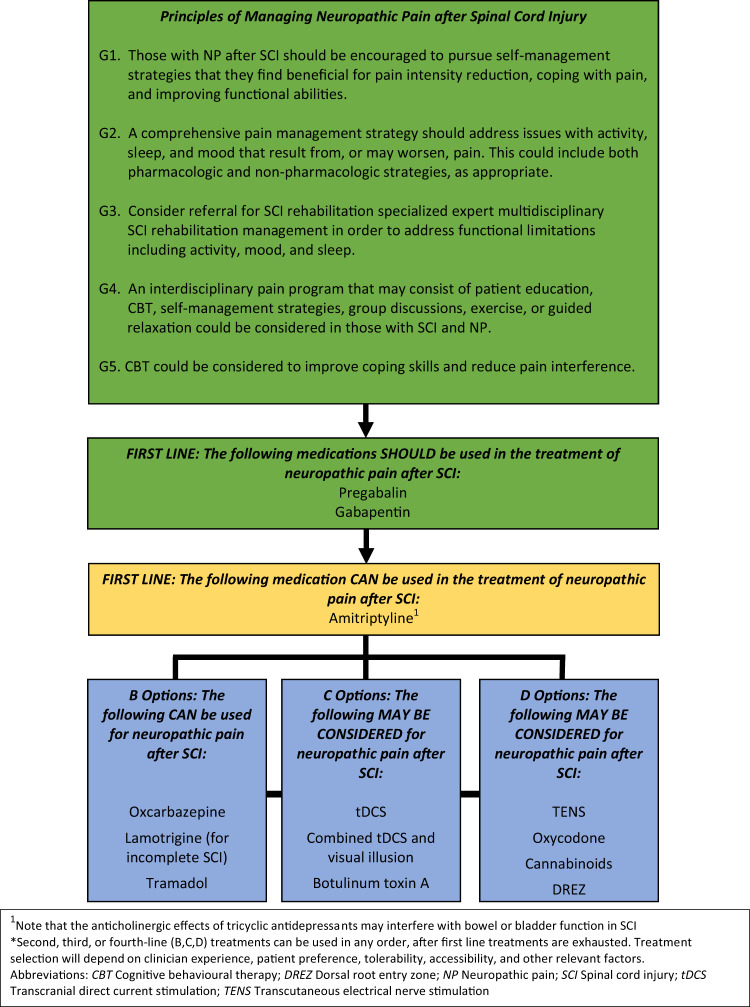
Principles of managing neuropathic pain after spinal cord injury. ^1^Note that the anticholinergic effects of tricyclic antidepressants may interfere with bowel or bladder function in SCI. *Second, third, or fourth-line (B,C,D) treatments can be used in any order, after first line treatments are exhausted. Treatment selection will depend on clinician experience, patient preference, tolerability, accessibility, and other relevant factors. CBT Cognitive behavioral therapy, DREZ Dorsal root entry zone, NP Neuropathic pain, SCI Spinal cord injury, tDCS Transcranial direct current stimulation, TENS Transcutaneous electrical nerve stimulation.

Although a hierarchy of treatment options was not used, the consensus panel agreed that classifying and phrasing the recommendations in this way would provide adequate clarity and information to the CPG user regarding the panel’s assessment and evaluation of different treatment options. Figure 1 presents the treatment recommendations as a diagram; Table 5 presents a full text listing all treatment recommendations.

### General treatment principles

#### 2021 new recommendations

***G1****.* Those with NP after SCI should be encouraged to pursue self-management strategies they find beneficial for pain intensity reduction, coping with pain, and improving functional abilities.

Type of evidence: expert opinion.

#### 2021 clinical considerations

The consensus panel acknowledges that the overall body of evidence for treatment of NP after SCI is limited. There may be self-management strategies that persons living with NP after SCI find beneficial for coping with their pain and improve functioning. These strategies may include hypnosis, exercise, yoga, massage, stretching, pacing education, peer support, and acupuncture. Evidence for these modalities, however, may be limited or non-existent [10–12]. If a person with SCI experiences improvement in NP and/or its impact on their daily life with these treatments and the treatments are acceptable to both the person receiving treatment and their health care provider, they should pursue the strategies that they find helpful.

***G2****.* A comprehensive pain management strategy should address issues that relate to, or may worsen, pain due to activity, sleep and mood interference. This could include both pharmacologic and non-pharmacologic strategies as appropriate.

Type of evidence: expert opinion.

#### 2021 clinical considerations

Sleep and mood are important aspects of chronic pain management in general. Sleep and mood can be worsened by pain, and vice versa. It is important to address sleep and mood with both pharmacologic and non-pharmacologic management strategies; clinical guidelines for the management of insomnia and depression can be consulted if needed [13–16]. It should be noted that some medications used for NP management in SCI may improve sleep (e.g., gabapentin, pregabalin, and amitriptyline). This may be a useful effect in some cases but would also require monitoring for excessive daytime sedation.

***G3****.* Consider referral to an SCI rehabilitation expert for specialized interdisciplinary management of functional limitations and associated issues of mood, activity and sleep.

Type of evidence: expert opinion.

#### 2021 clinical considerations

NP intensity may not significantly change despite best available management and may continue to significantly interfere with daily function. Assessment and management by an interdisciplinary team may provide improvements in function through adaptive strategies (including adaptive equipment) and education. Due to the specialized nature of SCI care and the unique needs of this population, referral to an SCI rehabilitation expert should be considered to address functional concerns associated with NP.

***G4****.* An interdisciplinary pain program that may consist of patient education, cognitive.

behavioral therapy (CBT), self-management strategies, group discussions, exercise, and other modalities could be considered in those with SCI and NP.

Type of evidence: expert opinion.

#### 2021 clinical considerations

Depending on the resources and expertise available in different clinical contexts, referral to a specialized interdisciplinary pain program could also be considered. Resources that are available within an interdisciplinary pain program may not be available in a specialized SCI rehabilitation clinic, and vice versa. Based on local factors, the needs of the person experiencing pain after SCI, and the clinical judgment of the provider, there may be benefit in accessing an interdisciplinary pain program and/or management by a specialized SCI interdisciplinary team.

***G5****.* CBT could be considered to improve coping skills and reduce pain interference.

Type of evidence: expert opinion.

### 2021 clinical considerations

5 articles [17–21] were identified that evaluate the impact of CBT on pain intensity; only one pre-post study [17] reported decreased pain intensity immediately following treatment and at 12-month follow-up (although no changes were seen at 6 and 9 months). Although the available evidence has not clearly supported the use of CBT to reduce NP intensity after SCI, it may reduce interference from NP after SCI, with associated improvements in sleep and mood [17–21]. The panel considers CBT an important aspect of a comprehensive management plan for those with NP after SCI, as pain intensity reduction can be elusive, especially if first-line options are not successful. CBT provides a means of potentially improving quality of life despite the presence of ongoing NP. This treatment could also be offered in conjunction with first-line treatments to improve coping and function, or as an option for first-line treatment if the patient prefers non-pharmacologic management, or if there is a contraindication to other first-line options.

### Specific treatment options

The following sections present first-line treatments, B, C and D options, and “advise against use” recommendations made by the WG. Within each category, the information is organized as follows:

1. New recommendations: Any new recommendations for the 2021 CPG are listed first, with clinical considerations that provide context for these recommendations immediately following the recommendation (listed as “2021 Clinical Considerations”).
2. Existing recommendations: Unchanged recommendations from the 2016 CPG are listed under this heading. “Clinical Considerations” accompanying each recommendation were included in the 2016 CPG to provide additional context and relevant clinical information. These “Clinical Considerations” are provided in this update as they appeared in 2016 (“2016 Clinical Considerations”). Additional context relating to any newly identified studies are provided in the section “2021 Update”. Although the WG did not formally evaluate effects on pain interference, these outcomes were discussed if those outcomes were available in the literature. Effects on pain interference are noted in the “2021 Update” section as appropriate, as they were not specifically included in the 2016 version.

### First-line treatments

First-line treatments have a high GRADE of evidence and a strong recommendation from the panel.

#### New recommendations

No new first-line treatment recommendations were proposed.

#### Existing recommendations

**Table:** 

Recommendation 2.1	
Pregabalin should be used for the reduction of neuropathic pain intensity among persons with spinal cord injury.	
Quality of evidence	High
Strength of recommendation	Strong

#### 2016 clinical considerations

Pregabalin is recommended as the first choice of first-line medications, as it has the strongest evidence of any treatment modality in below-level NP: all studies demonstrate a significant reduction in pain intensity. Pregabalin studies used larger sample sizes than most treatment studies for SCI-related NP and rigorous methodology. Two high-quality randomized placebo-controlled trials (RCTs) [22, 23] and one moderate-quality placebo-controlled RCT [24], which was downgraded based on wide confidence intervals around numbers needed to treat, comprise the evidence base for pregabalin. When defining successful treatment of individual patients as a 50% reduction in pain level, the NNT for these 3 trials was 7.1 [22], 7.0 [25], and 3.3 [24]. All studies focused on NP and measured pain intensity. One trial studied a mixed population that included stroke patients (*n* = 19), but a subgroup analysis was performed for the group with SCI [24]. A pharmaceutical company funded two pregabalin RCTs [22, 23]. Pregabalin studies used flexible dosing between 150 and 600 mg/day. Adverse effects include somnolence, dizziness, and edema which were usually of mild-to-moderate intensity and transient [22, 23, 26].

#### 2021 update

Two low/very low quality crossover studies [27, 28] did not demonstrate a difference between gabapentin and pregabalin for pain intensity reduction (*n* = 30 [29]; *n* = 28 [27]). Another high quality crossover study [30] (*n* = 55) showed pregabalin was significantly more effective than oxcarbazepine for those with evoked pain character of allodynia and heat hyperalgesia, but no difference in pain reduction between pregabalin and oxcarbazepine for those with evoked pain with character of electrical, burning, pricking, and numbness, or in those without evoked pain. A meta-analysis involving members of the SC from 2016 demonstrated significant decreases with pregabalin on pain (standardized mean difference: 1.71 ± 0.13; 95% CI, 1.458–1.965; *P* < 0.001). sleep interference, anxiety and depression [31]. No changes to the existing recommendation were proposed based on the newly evaluated studies.

**Table:** 

Recommendation 2.2	
Gabapentin should be used for the reduction of neuropathic pain intensity among persons with spinal cord injury.	
Quality of evidence	High
Strength of recommendation	Strong

#### 2016 clinical considerations

Gabapentin is recommended as the next choice when pregabalin is not an option or has been proven ineffective, as the evidence supporting gabapentin in SCI-related NP is not as strong as that for pregabalin. The body of evidence for gabapentin in SCI-related NP contains three randomized trials, two of which found no significant difference between gabapentin and placebo [32, 33], although one trial [33] found a trend toward pain intensity reduction with gabapentin (*n* = 7). The third study found gabapentin significantly reduced NP [34]. Two observational case series included only patients with SCI-related NP. One found a reduction in NP intensity [35], and the other found a reduction in (general) pain intensity [36]. An observational study of gabapentin in patients with SCI and different durations of symptoms, which included patients with cauda equina, found a significant reduction in the mean pain intensity score after treatment with gabapentin [37]. Maximum gabapentin doses in clinical trials ranged from 1800 to 3600 mg/day, and the major adverse events reported were dizziness and somnolence [32–34].

#### 2021 update

In the 2016 version, outcomes of reviewed studies were conflicting, but a meta-analysis performed as part of the data review indicated a significant decrease in pain with gabapentin (standardized mean difference = 1.20 ± 0.16; 95% CI, 0.88–1.52; *P* < 0.001) [31]. Additionally, in 2016, the panel agreed that further study was required to establish that gabapentin and pregabalin are interchangeable within the SCI population; it was noted that other CPGs for the management of central or peripheral NP consider both pregabalin and gabapentin first-line therapy. Two crossover trials were reviewed as part of the 2021 update, one of low quality [28] and the other of very low quality [27]. These studies reported similar reductions in pain intensity between pregabalin and gabapentin. Although these studies were not superior in quality to those previously included, they provide further evidence that both gabapentin and pregabalin reduce pain and pain interference in those with SCI and NP. The WG noted anticonvulsants, including gabapentin and pregabalin, do not impair motor recovery. These medications may have the most beneficial effect on motor recovery if given early.

Gabapentin may have positive effects on sleep; sleep interference scores improved after 8 weeks of treatment with gabapentin in one pre-post study, whether pain was present for less than, or equal to and greater than, 6 months [37].

**Table:** 

Recommendation 2.3	
Amitriptyline can be used for the reduction of neuropathic pain intensity among persons with spinal cord injury.	
Quality of evidence	High
Strength of recommendation	Strong

#### 2016 clinical considerations

If pregabalin and gabapentin have been ineffective, amitriptyline is recommended; less evidence exists for the efficacy of amitriptyline than for the gabapentinoids. A meta-analysis of four antidepressant RCT for the management of SCI-related NP found that these agents were effective in reducing NP [38]. Two RCTs that studied amitriptyline had conflicting results [32, 39]. One small study of patients with NP (*n* = 22) found amitriptyline more effective than active control (diphenhydramine) and gabapentin [32]. This study also found that NP was more likely to improve in patients with depressive symptoms. The second study of patients with NP (*n* = 51) and musculoskeletal pain (*n* = 33) found no significant difference between amitriptyline and control (benzotropine mesylate) [39]. Amitriptyline is typically used to treat NP at a dose of 25–150 mg/day [40]. Treatment is usually initiated at 10–25 mg daily. In the Rintala et al. study, which demonstrated amitriptyline efficacy in the treatment of SCI-related NP, nearly all participants reached the target dose of 50 mg three times a day [32]; these findings suggest that lower doses may be less effective. Adverse effects of TCAs include anticholinergic side effects, sedation, and cardiotoxicity, which mandate caution in the SCI population. Within other pain populations, secondary amine TCAs (nortriptyline and desipramine) tend to have similar efficacy but better tolerability than tertiary amines (amitriptyline and imipramine). There is a lack of evidence specific to patients with SCI-related NP for both secondary and tertiary amine TCAs.

#### 2021 update

A new randomized trial of moderate quality [41] was reviewed (*n* = 147) that compared amitriptyline to lamotrigine. No significant difference was seen between these treatments, although both amitriptyline and lamotrigine demonstrated statistically significant reductions in overall NP. The duration of this trial was relatively short (3 weeks), so it is not possible to comment on side effects that may be more evident with prolonged use. Additionally, the majority of participants in this study were younger (74% were 18–40 years old) and may therefore better tolerate amitriptyline. The panel continues to emphasize that amitriptyline can cause significant issues in those with SCI given its anticholinergic properties; for this reason, the recommendation for amitriptyline is that it “can be used” for the management of NP after SCI, rather than “should be used”.

### “B” options

These options were previously classified as “second-line” treatments. They represent either high or moderate evidence on GRADE and a “strong” strength of recommendation from the panel.

#### New recommendations

**Table:** 

Recommendation 2.4	
Oxcarbazepine can be used for the reduction of neuropathic pain intensity after spinal cord injury.	
Quality of evidence	High
Strength of recommendation	Strong

#### 2016 clinical considerations

A single high-quality randomized crossover study [30] compared oxcarbazepine and pregabalin. For those with evoked pain present, both medications demonstrated significant effect for electrical pain, burning pain, pricking pain, numbness, allodynia, and pressure analgesia. Oxcarbazepine did not have a significant effect on heat hyperalgesia, but pregabalin did. For those in the evoked pain absent group, both treatments demonstrated significant effect for electrical pain, burning pain, pricking pain, and numbness. As described in the pregabalin 2021 update section, there was no difference between oxcarbazepine and pregabalin in those with evoked pain present for electrical pain, burning pain, pricking, and numbness, however, pregabalin was significantly more effective than oxcarbazepine for those with allodynia and heat hyperalgesia. No significant difference was seen between treatments in those without evoked pain. Those with psychiatric diseases were excluded, so pharmacodynamic drug-drug interactions (particularly with antidepressant medications that can also cause the side effect of hyponatremia) for this subpopulation within SCI cannot be defined. When the severity of side effects associated with oxcarbazepine is compared to lamotrigine (which was already a “B” option) the side effect profile of oxcarbazepine is favorable. When considering this and the results of the crossover study, the panel agreed that oxcarbazepine could be used for NP after SCI. Despite the decreased severity of side effects associated with oxcarbazepine in the anticonvulsant class, the prescriber should be aware of several medication risks. There is a risk of hyponatremia (2–3%) especially within the first 3 months [42]; concomitant use with a selective serotonin reuptake inhibitor (SSRI) or diuretic medications requires close monitoring as these class of medications can also induce hyponatremia. Monitoring of serum sodium levels may be required at baseline and periodically while on treatment when patients have pharmacodynamic risk factors for hyponatremia. As a weak inducer of CYP3A4, those on oral contraception should be counseled that the medication could reduce the effectiveness of their contraceptive and additional contraceptive precautions (e.g., condoms) may be desired.

#### Existing recommendations

**Table:** 

Recommendation 2.5	
Tramadol can be used for the reduction of neuropathic pain intensity among persons with spinal cord injury.	
Quality of evidence	Moderate
Strength of recommendation	Strong

#### 2016 clinical considerations

A single randomized, placebo-controlled trial found a significant reduction in pain intensity with tramadol compared with placebo, but the evidence quality was downgraded because of wide confidence intervals [43]. The Canadian Guideline for Safe and Effective Use of Opioids for Chronic Non-Cancer Pain is a useful resource for general information on opioid management and prescription considerations [44]. Although tramadol is not a scheduled drug in Canada at the time of writing this CPG, Health Canada is currently reviewing tramadol for a potential scheduling change and is listed as a Schedule IV drug in the United States. The maximum daily dosage of tramadol is 400 mg [40]. Common adverse effects are sedation, nausea and constipation. Twelve out of thirteen participants in the Norrbrink and Lundeberg [43] trial withdrew because of adverse medication events. A slight increase in the risk of serotonin syndrome can be seen when tramadol is combined with other serotonergic drugs such as SSRIs, serotonin norepinephrine reuptake inhibitors, TCAs, etc [45].

#### 2021 update

No new studies were identified for review. The WG reviewed pain interference descriptions in the literature, and no significant changes in pain interference were noted with tramadol compared to placebo [43].

**Table:** 

Recommendation 2.6	
Lamotrigine can be considered in those with incomplete spinal cord injury for the reduction of neuropathic pain intensity.	
Quality of evidence	Moderate
Strength of recommendation	Strong

#### 2016 clinical considerations

Evidence for the efficacy of lamotrigine has been demonstrated only in patients with an incomplete SCI. As a result, lamotrigine is recommended as second-line therapy only in this subpopulation. One randomized placebo-controlled trial showed lamotrigine significantly reduced the intensity of NP for patients with incomplete SCI; the evidence quality was downgraded because of wide confidence intervals [46]. Lamotrigine was titrated to a maximum dose of 400 mg per day [46]. Common adverse effects were dizziness, somnolence, headache, and rash. It should be noted that lamotrigine has a black box warning issued by the United States Food and Drug Administration for serious skin rashes, including Stevens-Johnson Syndrome.

#### 2021 update

One additional randomized longitudinal study [41] of moderate quality evaluated the comparative effect of lamotrigine and amitriptyline. This study included both patients with complete and incomplete SCI, and although both amitriptyline and lamotrigine demonstrated statistically significant reductions in overall NP, there were no significant between-group differences for overall NP at any follow-up time point. Follow-up was within a relatively short time period of 3 weeks, so the long-term effectiveness of these treatments was difficult to ascertain from this study. The majority (76%) of participants in this study were classified as an American Spinal Injury Association Impairment Scale (AIS) grade A lesion. The expert panel therefore discussed the potential use of lamotrigine in those with complete SCI. As follow-up was for a short period and treatment was unblinded, the panel decided there was not enough new evidence to support a change in the recommendation. The panel concluded the effect of lamotrigine on NP in those with complete SCI requires more research.

### “C” options

These options were previously classified as “third-line” treatments. They represent either high or moderate evidence on GRADE and a “weak” strength of recommendation from the panel.

#### New recommendations

**Table:** 

Recommendation 2.7	
Botulinum toxin A may be considered in the management of below-level neuropathic pain after SCI, with injection localized to the area of maximal pain.	
Quality of evidence	High
Strength of recommendation	Weak

#### 2021 clinical considerations

One RCT of high quality has been conducted to evaluate the effect of botulinum toxin A on NP intensity in patients with SCI [47]. This study found significant reductions in pain intensity compared to placebo at 4 and 8 weeks following injection for below-level (but not at-level) NP. The sample size in this study was relatively small, and the findings of this study require replication. Further research is required to determine whether other factors, such as, level and completeness of injury, may affect treatment efficacy. The panel also recognizes access to this treatment may be limited as it is an off-label indication, and may present a barrier to obtaining drug coverage. Given the evidence for botulinum toxin A is preliminary for NP, the expert panel felt that only a weak recommendation was appropriate.

#### Existing recommendations

**Table:** 

Recommendation 2.8	
Transcranial direct current stimulation (tDCS) may be considered for reducing neuropathic pain intensity among persons with spinal cord injury.	
Quality of evidence	High
Strength of recommendation	Weak

#### 2016 clinical considerations

tDCS is recommended as “C” therapy for patients with SCI-related NP based on four RCTs. Three studies found a significant reduction in pain intensity with tDCS compared to sham control [48–50]. One of these studies found a significant improvement in continuous pain on the last day of treatment, and paroxysmal pain at follow-up, but no significant reduction in overall pain intensity [50]. A fourth study did not find a significant difference between tDCS and sham control [51]. A prospective controlled trial that found a significant reduction in pain intensity, compared to sham control, was upgraded because of small confidence intervals and the inclusion of an intention-to-treat analysis [52]. In our prior meta-analysis of the five studies we found a positive effect for tDCS on pain intensity (SMD = 0.510 ± 0.202; 95% CI, 0.114–0.906; *p* = 0.012) [53]. tDCS was, however, given a weak strength of recommendation, as the effects were not maintained over time and the panel felt it was more appropriate to trial pharmacological therapies first, in accordance with other NP management guidelines and extensive clinical experience with those treatments in NP secondary to various etiologies. Minor side effects of tDCS include skin irritation, which can be minimized by preparing electrodes with saline and the skin with electrode cream and by increasing current gradually. Another side effect, phosphene, which is the visual perception of a brief flash of light, can be avoided with correct electrode placement (not placed too close to the eye).

#### 2021 update

One RCT [54] of low quality provides additional supporting evidence for NP intensity reduction following tDCS in comparison to sham control, while one RCT crossover of moderate quality did not find a significant difference in NP intensity between active and sham tDCS treatments [55]. An RCT crossover of very low quality examined a single session of active or sham tDCS, neither of which resulted in reductions in pain intensity following treatment [56]. One low quality pre-post study found a significant decrease in pain intensity following tDCS treatment at 2-week follow-up, although this was not sustained at 3 weeks [57]. From a pain interference perspective, tDCS improved mood, general activity and the ability to get around on the last day of treatment (day 14) [50].

A meta-analysis of all nine studies found a positive short-term effect for tDCS on pain intensity, indicating a beneficial effect of treatment that is limited in duration. The expert panel noted that there is variability in treatment protocols for tDCS in different studies, which makes generalizability difficult. No changes were made to the recommendation at this time.

**Table:** 

Recommendation 2.9	
Combined visual illusion and transcranial direct current stimulation may be considered for reducing neuropathic pain intensity among persons with spinal cord injury.	
Quality of evidence	High
Strength of recommendation	Weak

#### 2016 clinical considerations

An RCT in SCI-related NP found a significant reduction in pain intensity after treatment compared to control illusion, visual illusion in isolation, and tDCS in isolation [50]. An observational study of a cohort that included individuals with neuropathic and other types of pain found a nonsignificant improvement in pain intensity after treatment [58]. The main side effects of this combined therapy included mild headache and fatigue [50].

#### 2021 update

No new studies were identified for review. Evaluating previously included studies from a pain interference perspective, combined tDCS and visual illusion resulted in improvements on various aspects of the Brief Pain Inventory at various timepoints after initiating treatment when compared to tDCS alone, visual illusion alone, or placebo, at various time points [50].

### ”D” options

These options were previously classified as “fourth-line” treatments. They represent either moderate or low evidence on GRADE and a “weak” strength of recommendation from the panel.

#### New recommendations

**Table:** 

Recommendation 2.10	
Cannabinoids may be considered for the management of neuropathic pain among persons with spinal cord injury.	
Quality of evidence	Moderate
Strength of recommendation	Weak

#### 2021 clinical considerations

Two studies have been conducted to evaluate the effect of cannabinoids on NP intensity in patients with SCI [59, 60]. One RCT crossover of moderate quality investigated two different strengths of vaporized cannabis (i.e., 2.9 or 6.7% delta-9-tetrahydrocannabinol), and found that pain intensity was significantly lower for both compared to placebo, with no difference in analgesic effect between the active doses [59]. The follow-up period for outcome measurement in this study was only 7 h. There was a lack of graded analgesic response with higher doses. Blinding was a concern in this study given that psychoactive effects had a dose dependent response. An earlier pilot RCT of low quality did not find a difference in NP intensity reduction between dronabinol and active control (diphenhydramine) groups [60].

The panel also discussed evidence for the effectiveness of cannabinoids (including nabiximols) in other central NP conditions besides SCI. The expert panel agreed that the adverse effect profile for cannabinoids was preferable to that of oxycodone and other opioids. Risks of tolerance and hyperalgesia associated with opioids also made the option of cannabis potentially advantageous as a treatment choice. Therefore, the expert panel chose to include a specific recommendation regarding cannabis for NP after SCI, although further research regarding its effects is still needed.

#### Existing recommendations

**Table:** 

Recommendation 2.11	
Transcutaneous electrical nerve stimulation (TENS) may be considered for the reduction of neuropathic pain intensity among persons with spinal cord injury.	
Quality of evidence	Low
Strength of recommendation	Weak

#### 2016 clinical considerations

One prospective controlled observational study has evaluated the effect of TENS in patients with SCI-related NP [61]. This trial found no significant difference between high-frequency and low-frequency TENS. An early (1975) observational case series found a pain intensity reduction with TENS for two of 11 patients [62]. The evidence quality of this study was downgraded because of a lack of confidence intervals and description of methods, and a potential for bias.

It is important to consider the short duration of action for relief of pain with TENS when contemplating use of this modality. In addition, lack of long-term follow-up precludes any discussion of the prolonged efficacy of TENS. Few side effects are associated with TENS, although patients have reported increased pain intensity and muscle spasm [63]. The WG did not include TENS among the therapies without specific recommendations because of a lack of long-term follow-up, however, the relatively innocuous side effects of TENS make this therapy appropriate for a therapeutic trial in refractory cases.

An additional consideration is electrode placement. Although evidence is limited, the utility of TENS when electrodes are placed in insensate areas has been demonstrated. Recent trials have used placement of electrodes at the level of injury in an area with preserved or intact sensibility [62, 63].

#### 2021 update

Three studies provide additional supporting evidence for NP intensity reduction following treatment with TENS. One RCT crossover study of moderate quality compared TENS with visual illusion [64]. A significant reduction in pain intensity was found after TENS application for 2 weeks, while there was no significant decrease in pain intensity after 2 weeks for visual illusion. On the BPI, TENS significantly decreased the effect of pain on mood, sleep and relationships with others; visual imagery improved walking ability [64]. In another RCT of moderate quality, low frequency TENS was found to significantly reduce pain intensity compared to baseline after 10 days of treatment, while no significant effect was observed with sham TENS [63]. This study was originally excluded in the 2016 version, but upon review, it was found to fit criteria for the 2021 update and was reviewed again for this update. A pre-post study of very low quality showed a significant reduction in pain intensity compared to baseline following 8 weeks of treatment with high frequency TENS [65]. A meta-analysis of four studies found a positive effect for TENS on pain intensity [61, 63–65]. The expert panel determined that the new evidence would not significantly strengthen or weaken the previous recommendation or affect it negatively. Thus, this recommendation is unchanged.

**Table:** 

Recommendation 2.12	
Oxycodone may be considered for the reduction of neuropathic pain intensity among persons with spinal cord injury.	
Quality of evidence	Moderate
Strength of recommendation	Weak

#### 2016 clinical considerations

Oxycodone is an oral opioid that has been assessed in SCI-related NP. One observational study, which showed a significant reduction in pain intensity after 3 months of treatment, had its quality of evidence upgraded because confidence intervals were provided and only patients with NP were included [66].

Long-term opioid use exposes individuals to unique risks including possible problems with drug tolerance and dose escalation, physical dependence, opioid induced hyperalgesia, endocrinopathy and potential for misuse/addiction [67]. Typical opioid adverse effects include sedation, nausea, vomiting, constipation and dry mouth [44]. Constipation in particular can be problematic in people with SCI who may have pre-existing neurogenic bowel changes. Oxycodone is around approximately one and a half times as potent as morphine. The potential adverse effects and issues associated with oxycodone, and opioids in general, led the panel to assign a weak strength of recommendation. The panel recommends additional research into the use of opioids as a class in patients with SCI-related NP. It is likely reasonable to use opioids other than oxycodone, and the Canadian Guideline for Safe and Effective Use of Opioids for Chronic Non-Cancer Pain should be used to guide the use of medications in this class [44].

#### 2021 update

No new studies were identified for review.

**Table:** 

Recommendation 2.13	
The DREZ procedure may be considered in exceptional circumstances and as a last resort for reducing neuropathic pain intensity among persons with spinal cord injury.	
Quality of evidence	Low
Strength of recommendation	Weak

#### 2016 clinical considerations

Evidence of benefit for the DREZ procedure exists, but the risk of the procedure does not justify its use beyond exceptional circumstances. The available evidence supporting the DREZ procedure is based on observational studies or case series in SCI-only populations [68–72], with one study including patients with cauda equina [70]. A prospective-controlled observational trial found a reduction in pain intensity with the DREZ procedure and greater efficacy in below-level pain [68]. An observational study found a reduction in pain intensity after the DREZ procedure [70]. Three observational case series found the DREZ procedure reduced pain intensity [70–72]. Risks associated with the DREZ procedure include paresis, neuropathy or radiculopathy, ataxia, sensory loss, and a variety of surgical complications, such as persistent incisional site pain, cerebrospinal fluid leak, wound infection, subcutaneous hematoma, and bacteremia [69, 71].

#### 2021 update

Two pre-post studies of very low quality provide additional supporting evidence for NP intensity reduction following the DREZ procedure. One study found pain relief 2 weeks and 3 months after the procedure, as well as at long-term follow-up (average of 36.1 months) [73], while the other study found complete or near-complete below-level NP relief following the DREZ procedure at follow-up periods ranging from 1.5 to 11 years [74]. The risk for DREZ remains the same, and the level of evidence is unchanged from the previous version of the guidelines at “low”. The recommendation was unchanged in this version.

### Advise against use

The studies for these treatment options showed no effect or benefit in the SCI population, and therefore recommendations advise against their use.

**Table:** 

Recommendation 2.14	
Levetiracetam should not be used for reducing neuropathic pain intensity among persons with spinal cord injury.	
Quality of evidence	High
Strength of recommendation	Strong

#### 2016 clinical considerations

A prospective RCT performed in a population with SCI-related NP comparing levetiracetam to placebo found no significant difference between the two treatments [75].

#### 2021 update

No new studies were identified.

**Table:** 

Recommendation 2.15	
Mexiletine should not be used for reducing neuropathic pain intensity among persons with spinal cord injury.	
Quality of evidence	High
Strength of recommendation	Strong

#### 2016 clinical considerations

A prospective, placebo-controlled RCT in a population of patients with SCI-related NP found mexiletine was not significantly more effective than placebo in reducing pain intensity [76].

#### 2021 update

No new studies were identified.

### Therapies requiring further research

For various reasons, the WG did not make specific recommendations for some treatments that were previously evaluated for the original 2016 CPG. Insufficient evidence for benefit, studies with conflicting results and insufficient data to perform meta-analyses, lack of enough studies to provide pooled data, lack of evidence of long-term effect or follow-up, evidence of positive effect in populations other than SCI, or low-quality studies with negative results may have resulted in a “requires further research” designation from the WG. The only treatment that moved from “requires further research” to a specific recommendation was cannabinoids, which is now a “D” option.

Additionally, the WG identified 14 new treatment options, not evaluated in the 2016 version, as requiring further research. Descriptions of these treatments are provided in this section. The WG also re-evaluated treatments that were previously designated as requiring further research in the 2016 CPG if new studies were available for review. These included hypnotic suggestion, transcranial magnetic stimulation, intravenous (IV) ketamine, visual illusion, and acupuncture. Updated descriptions for these treatments are provided below; the panel did not make new recommendations for these management options. No new studies were identified for exercise, spinal cord stimulation, massage, osteopathy, duloxetine, IV lidocaine, IV alfentanil, IV clonidine and morphine, or IV morphine. The previous descriptions of these treatments from the 2016 version of the guidelines are included within this document to provide a single, updated resource.

#### New treatments—further research required

The treatments in this section were identified as requiring further research and were not previously reviewed in 2016.

#### Intermittent normobaric hyperoxia

A single moderate quality RCT (*n* = 62) found that a group receiving 4 h of pure oxygen had significant improvement in the mean visual analogue scale score at 14 days compared to a group receiving 4 h of air (control) and 3 h of air plus 1 h of pure oxygen (group A) [77]. No long-term effect (day 30 and day 60) was seen, although data was not provided. Given the lack of long-term benefit, no recommendation was made.

#### Neurofeedback training

Three studies of very low quality (one RCT crossover, and two pre-post studies) evaluated neurofeedback training. The pre-post studies demonstrated some benefit, although sample sizes were small [78, 79]. The RCT crossover demonstrated no benefit of a single session of neurofeedback training on pain intensity [56]. Evidence was insufficient to make a recommendation at this time.

#### Anti-inflammatory diet

A single moderate quality RCT showed a significant group versus time interaction on the anti-inflammatory diet for the sensory component of the self-reported NP questionnaire [80]. Post hoc analyses revealed a significant reduction in sensory scores in the anti-inflammatory diet group from baseline to 1 month and 3 months. The WG noted that the study suggests a possible relationship between changes in inflammatory mediators and sensory scores; findings should be replicated in a larger study.

#### Ultramicronized palmitoylethanolamide (PEA-um)

One high-quality RCT showed no benefit with ultramicronized palmitoylethanolamide compared to placebo [81]. Although there was no evidence to suggest that it should be used, the WG determined there was not enough evidence to make a recommendation against its use. Timing of administration may be important, and earlier administration post injury may demonstrate a more favorable outcome. The WG noted PEA-um has a positive effect in other NP populations, and would therefore benefit from additional study.

#### Breathing-controlled electrical stimulation (BreEStim)

One high-quality [82] and one moderate-quality [83] RCT crossover study demonstrated a treatment benefit. The WG was concerned regarding the resource burden of the treatment. The findings were considered too preliminary for a recommendation to be made.

#### BreEStim + tDCS

One moderate quality RCT crossover did not find a difference between combined tDCS and BreEStim treatment and BreEStim treatment alone [55]. In consideration with studies examining BreEStim alone, this study did not significantly change the overall quality of evidence to warrant a recommendation for BreEStim.

#### Neurotensin (CGX-1160)

The WG agreed one low-quality study [84] with a sample size of four was not sufficient to justify a recommendation.

#### Autologous mesenchymal stromal cells

Four pre-post studies of very low quality of autologous mesenchymal stromal cells (MSC) demonstrated mixed results, with three studies demonstrating decreased pain up to 10 months post MSC transplantation [85–87], and one study demonstrating no significant difference in pain intensity 6 months post treatment [88]. Additional studies are needed to confirm benefit.

#### Neuromuscular electrical stimulation + carbamazepine

One prospective controlled study of very low quality investigating combined neuromuscular electrical stimulation and carbamazepine did not demonstrate additional benefit for the combined treatment over the group that received carbamazepine alone [89]. The WG concluded a recommendation could not be made either way based on one very low quality study.

#### Intrathecal baclofen

A moderate-quality RCT demonstrated improvement in NP pain intensity after intrathecal baclofen 4 and 8 h post treatment compared to the placebo group [90]. There was also significant improvement in pain interference with mood, general activity, moving around, enjoyment of life, ability to work and perform daily tasks, and social relationships in the treatment group. Additional evidence, including long-term follow-up, is required for a recommendation.

#### Meditation

One crossover trial of very low quality evaluating meditation and other non-pharmacologic treatment modalities against sham tDCS showed a significant reduction in pain intensity for meditation compared to sham tDCS [56]. Follow-up measurement of pain intensity, however, was only evaluated up to 10 min after treatment. Given the very low quality of the study and the short duration of follow-up, no recommendation was made by the WG at this time.

#### TENS + gabapentin

A RCT of very low quality described significantly decreased pain intensity after 10 days of treatment with a combination of TENS and gabapentin compared to sham TENS and gabapentin [91]. There was insufficient evidence for a recommendation.

#### Overground bionic ambulation

One very low quality pre-post study of three participants evaluated overground bionic ambulation [92]. There is insufficient evidence for a recommendation to be made.

#### Venlafaxine

A single RCT found no significant improvement in pain intensity or interference outcomes for venlafaxine compared to placebo [93]. The WG voted on a possible recommendation *against* the use of venlafaxine for NP after SCI. 63% of WG members agreed that further research into this treatment would be appropriate, rather than advising against use. 6% of WG members suggested a potential fourth-line role for venlafaxine. The WG noted that although this study suggested no specific effect on central NP for venlafaxine, it may have a role in mixed neuropathic and nociceptive presentations; additional research is required to replicate these findings.

#### 2021 updates for previously evaluated treatments—further research required

The following section lists treatments requiring further research that were previously evaluated in 2016, and for which additional studies were identified for the 2021 CanPainSCI CPG update. These treatments continue to require additional research. The previous description for these treatments from the 2016 version is included, with a separate section describing the 2021 update.

#### Hypnotic suggestion

*2016:* An RCT found a reduction in intensity of SCI-related NP after treatment (*p*<0.01), but the evidence quality was downgraded because of a lack of confidence intervals [94].

*2021 update:* An additional crossover trial of very low quality evaluated hypnosis and other non-pharmacologic treatment modalities (e.g., meditation, described in previous suggestion) against sham tDCS [56]. There was significant reduction in pain intensity following this treatment, although follow-up was only continued for 10 min post treatment. The new study was not sufficient to change the requirement for further research into hypnotic suggestion.

#### Transcranial magnetic stimulation (TMS)

*2016:* Three RCTs in SCI-related NP compared the effect of TMS with sham therapy [95–97]. Two of these trials found no significant difference in reduction in pain intensity [95, 96]. The evidence quality of the third trial, which found a significant improvement in pain intensity after treatment, was downgraded because of wide confidence intervals [97].

*2021 update:* Two additional RCTs were identified. One RCT demonstrated no effect [29], while the other demonstrated a positive effect for up to 4 weeks only [98]. For pain interference, there was no significant difference between real and sham TMS on the BPI [96]. The WG voted on a potential recommendation for TMS, but the consensus threshold for a new recommendation was not met (61.5% agreed but 75% was needed to pass this as a new recommendation); additional study with longer-term follow-up is needed. The logistics of providing this treatment was also raised as a concern.

#### Acupuncture

*2016:* One study showed no significant effect on chronic pain intensity in patients with SCI-related pain or chronic musculoskeletal pain; non-responders were all from the central pain population [99]. In another study, eight of fifteen patients with SCI-related NP responded to acupuncture [100]. A retrospective observational case series of patients with traumatic or nontraumatic SCI found a significant improvement in pain for bilateral, symmetric, burning, or constant pain compared with unilateral, asymmetric, atypical, or intermittent pain [101]. Studies of acupuncture suffer from a lack of standardization of process or procedure delivery and practice principles, and evidence for effectiveness is inconclusive. Additional studies are needed to clarify the benefit of using this modality.

*2021 update:* A moderate quality crossover trial assessed battlefield acupuncture (BFA) against a waitlist control [102]. A significantly larger decrease in pain severity (NRS scores) was observed in the battlefield acupuncture group compared to the control group. The WG expressed concerns regarding the lack of a true control group (waitlist control group), and a specific methodology for delivering treatment (battlefield acupuncture). There was also a higher baseline pain intensity in the treatment group as compared to the control group. A second very low quality pre-post study examining percutaneous electrical nerve stimulation was also reviewed [103]. This study demonstrated significantly decreased pain intensity on the NRS at 8 and 18 weeks post treatment. One study [104] suggested that acupuncture may also have an effect on pain interference with activities of daily living at 7.5 weeks, but not at 3 months. The WG again discussed the large variation in practice and techniques for acupuncture, with the lack of a standardized protocol being problematic despite some evidence for effect. Some members noted there is evidence in the literature, and extensive practical experience, that suggests the treatment is safe. The WG voted on a possible recommendation for acupuncture. A slight majority (56%) agreed that acupuncture should be a recommendation, but this did not reach threshold for inclusion (75%).

#### Visual illusion

*2016:* One RCT in patients with SCI-related NP found a significant reduction in overall pain intensity compared with a control illusion on the last day of treatment, but this change did not persist and was not evaluated at follow-up [50]. An observational study that found an insignificant reduction in pain intensity with the intervention was downgraded because of a lack of information on sequence generation, wide confidence intervals, and a very small sample size (*N* = 5) [105]. Another observational study found a significant increase in pain intensity after treatment in comparison with a control illusion [106].

*2021 Update:* One moderate quality crossover study [64], one low quality RCT [107], one low quality pre-post study [32] and two additional very low quality studies [108, 109] were reviewed by the WG. The WG considered the new evidence in conjunction with the 2016 studies. Interventions differed widely between studies, which made comparison difficult. Some studies had no longitudinal evaluation, and it was difficult to evaluate long-term effect overall. The WG also raised questions regarding the mechanism of this treatment, and whether visual illusion had an impact on neuroplasticity, or was useful to serve only as a distraction from pain. The WG did vote on a potential recommendation for visual illusion, but only 61.5% agreed that it should be a recommendation, which was lower than the required threshold. Further research was therefore required before a recommendation could be made.

#### IV ketamine

*2016:* Two RCTs of IV ketamine in SCI-related NP found a significant reduction in pain intensity [110, 111]. The evidence quality of one study was downgraded because of the absence of a power calculation and an unclear protocol [110]. The second study, a double-blind crossover study of nine patients with central dysesthetic pain after SCI, evaluated ketamine and alfentanil compared with placebo (normal saline) [111]. Ketamine significantly reduced the intensity of continuous pain and allodynia compared with placebo. As the treatment response was only measured before and after infusion, the duration of response is uncertain.

*2021 Update:* No new study on IV ketamine was done, although a topical ketamine pre-post study of five people was evaluated [112]. Ketamine continues to require additional research in SCI NP.

### Previously evaluated treatments with no new studies—further research required

The following section lists treatments requiring further research that were evaluated in 2016, for which no additional studies were identified in the 2021 CanPainSCI CPG update. The 2016 description for these treatments is included in this section.

#### Exercise

One RCT of pain in patients with SCI found a reduction in the pain perception score in the treatment group compared with the control group after 3 months (*F* [1, 27] = 4.99, *p* = 0.03) [113]. However, the evidence quality of the study was downgraded because of the following: an unclear protocol; lack of specification of the type of pain, blinding, confidence intervals, and power calculation; and potential bias of control participants.

#### Spinal cord stimulation

A case series with a mixed pain population of patients with SCI presented no statistically significant data on pain intensity reduction [114].

#### Massage

A prospective-controlled trial, which included a comparison between acupuncture and massage, found that massage did not produce a significant reduction compared with acupuncture in SCI-related NP intensity [100]. No evidence was found on efficacy of massage on its own.

#### Osteopathy

An RCT found a 16% reduction in the perception of SCI-related NP during treatment but not at later time points [115]. No significance was reported for this result, and the evidence quality of this study was downgraded because of a lack of randomization process description, blinding and confidence intervals.

#### Duloxetine

One RCT showed no significant difference in reduction in intensity of NP between duloxetine and placebo in patients with SCI or stroke, although a trend was seen toward a decrease in the mean pain score with duloxetine, demonstrating the potential for benefit [116]. A good evidence base exists for the effectiveness of duloxetine in treating peripheral NP in other populations [117]. The CPG for the prevention and management of diabetic neuropathy recommends duloxetine as an option for the treatment of NP in this population [118]. Doses of 60 and 120 mg appear to be effective in reducing the intensity of peripheral NP. Nausea is a typical side effect. Clinically insignificant increases in blood pressure can occur while hepatotoxicity is rare [40].

#### IV lidocaine

IV lidocaine has demonstrated benefit in three well-conducted RCTs of SCI-related NP [110, 119, 120]. One of these trials also included patients with stroke [120]. Two of the studies found a significant reduction in pain intensity with IV lidocaine compared with placebo [119, 120], whereas the third found no significant difference [110]. The evidence quality of one study was downgraded because of a lack of power calculation and an unclear protocol [110], while another was downgraded because of a lack of explanation of the randomization process and large confidence intervals [119]. The studies with a positive result used lidocaine 5 mg/kg^−1^ over 30 min [119, 120], whereas the study with a negative result used lidocaine 2.5 mg/kg^−1^ over 40 min [110]. Light-headedness was a common adverse effect. As the duration of benefit for IV lidocaine is very short, this treatment modality should only be considered in specific circumstances where a short duration of effect is desired.

#### IV alfentanil

A single double-blind cross-over study of nine patients with central dysesthetic pain after SCI evaluated alfentanil and ketamine compared with placebo (normal saline) [111]. Alfentanil significantly reduced the intensity of continuous pain compared with placebo (*p* = 0.01), allodynia compared with placebo and wind-up-like pain compared with placebo. As the response of continuous pain to therapy was only measured before and after the infusion, the duration of response is uncertain. The IV mode of administration of alfentanil makes this therapy a short-term management option with a short duration of effect.

#### Intrathecal clonidine

One RCT that compared intrathecal clonidine with placebo (saline) found no statistically significant difference in reduction of pain intensity in patients with SCI-related pain, and the evidence quality of the study was downgraded because of a lack of description of the randomization process or patient allocation [121].

#### Intrathecal clonidine and morphine

One double-blind cross-over study of 15 patients that compared intrathecal administration of clonidine, morphine, clonidine plus morphine and saline (placebo) found a significant reduction in pain intensity only for the combination of intrathecal morphine and clonidine compared with placebo (*p* = 0.0084) [121]. Two of four patients with at-level pain and five of 14 patients with below-level pain responded to the combination, but no significant difference was found between the groups. As a significant correlation was seen between pain relief and drug concentrations in the cervical cerebrospinal fluid, consideration should be given to administering the agents above the level of injury to ensure adequate penetration of cervical cerebrospinal fluid. The duration of response is uncertain but is assumed to be 24 h, as patients were crossed over to the next therapy the following day.

#### IV morphine

One small, double-blind, placebo-controlled, cross-over study of IV morphine in patients with SCI- or stroke-related NP found no significant difference in spontaneous pain between placebo and IV morphine but a 90-min post-injection reduction in allodynia with morphine [122]. The evidence quality of the study was downgraded because of the lack of power calculation. All patients subsequently received oral sustained-release morphine, but the titration schedule was not well defined, and the study used haloperidol, an uncommon treatment for opioid-induced nausea. Overall, the results of this study were inconclusive for a benefit of IV morphine.

## Discussion

These guidelines represent the most up-to-date version of the CanPainSCI CPG. As in the 2016 CPG, the primary outcome for treatment options evaluated through the GRADE process was pain intensity. The WG is aware this is not the only relevant outcome, nor is it the most important outcome in all cases. Although not formally evaluated within this version of the CPG, the WG considered other outcomes (e.g., function, pain interference, etc.) in their discussions. Future editions of the CPG will formally assess other relevant pain outcomes using GRADE.

A limitation of the 2016 CPG was the lack of high-quality studies; it was recognized by the 2016 panel that further research is required for most treatment options including those that were developed into recommendations. In the intervening time since the initial 2016 CanPainSCI guidelines, the overall study quality on which recommendations are based has not drastically improved. Of the 43 new articles reviewed in the treatment category, all but five articles were considered moderate to very low in quality.

A case could certainly be made that the evidence is too weak for many of the CanPainSCI recommended treatments to be presented as specific recommendations. The CPG WG panel discussed this issue at length. The WG agreed that although the overall evidence base remains weak, it is unacceptable for those with NP after SCI, and for clinicians, to have no guidance on management. Although study quality is limited, extensive work has been done within NP and SCI, and the consensus of the CanPainSCI panel represents a best effort to summarize existing work and to develop a framework to guide management decisions. High quality trials of NP treatments after SCI can be challenging to produce given the low prevalence of SCI, which is further complicated by variations in etiology, level, and severity. As such, the WG noted that significant improvements in the overall quality of the evidence base could take years, if not decades, to achieve. It is, therefore, important to have a document that provides a framework for NP management after SCI, and the panel agreed on the importance of the guidelines to serve as a reference point for clinicians and to identify areas for research for NP after SCI.

The CanPainSCI panel made the decision to restructure the classification of treatment recommendations from first to fourth line to the current “first-line”, “B”, “C”, and “D” system, to account for the inherent challenges of working with a limited evidence base. An additional general recommendation regarding the pursuit of self-management strategies that those with SCI find successful (G1) was also included as a response to the limited evidence that underlies many treatment options.

### Update

The process of updating the CanPainSCI CPG was discussed by the panel. While formal updates are intended to occur every 4–5 years, the panel noted it may be less onerous to conduct smaller updates more frequently, and would keep the CPG continually up-to-date. A living guidelines process was discussed, with updates and reviews to be performed on a yearly basis, involving smaller, rotating groups within the WG. The full panel would review any changes at the 4–5-year point as scheduled, and issue a formal update of the CPG at that time. We expect the living guideline process to begin at the end of 2021.

### Applicability

The next steps for implementation of the CPG will be refinement of existing clinical algorithms to allow easier uptake at point-of-care. Alternative means of disseminating the guidelines, including via phone app, are in development. Appendix 3 provides a visual summary of the algorithm in development for the screening, diagnosis, and treatment of NP following SCI. While measuring the impact of the CPG on specific, quantifiable pain outcomes is challenging given the considerable flexibility in selecting treatment options, a project is underway to implement the CanPainSCI CPG within a local context to identify barriers and facilitators for a broader national implementation strategy.

## Supplementary information

Appendix 1 - Search Strategy

Appendix 2 - GRADE scoring criteria

Appendix 3 - Visual representation of a clinical algorithm for the screening/diagnosis and treatment of neuropathic pain following SCI
