# Supplementary material for: The CanPain SCI clinical practice guidelines for rehabilitation management of neuropathic pain after spinal cord injury: 2021 update
Source: Spinal Cord. 2022 Feb 5;60(6):548–66. doi: 10.1038/s41393-021-00744-z (PMC9209331; doi:10.1038/s41393-021-00744-z)
Supplement: Supplementary file 1 — Appendix 1 - Search Strategy [file 41393_2021_744_MOESM1_ESM.docx]

| Step | Details |
| --- | --- |
| 1. Literature search | SCI-related NP |
| 1. Databases | MEDLINE, EMBASE, CINAHL, PsycInfo, Cochrane library (Central Register of Controlled Trials) |
| 1. Key words* | Spinal cord injur*  AND  neuropathic pain OR pain OR central pain  AND  treatment OR intervention* OR therapeutic OR diagnos* OR classification system* OR outcome measure* OR assessment tool* OR models of care OR model of care OR healthcare delivery |
| 1. Limits | English, 2013-2014, 2015-October 2018 |
| 1. Inclusion criteria | Articles which investigated interventions for the treatment of neuropathic pain in people with spinal cord injury were included if they met the following inclusion criteria:  traumatic or non-traumatic SCI etiology; human adult study participants ≥18 years old; study population ≥50% SCI; study population NP or mixed pain; N ≥ 3 participants (SCI + NP/mixed pain); rehabilitation, outpatient, or community setting/non-acute chronicity; any treatment intervention; effect of treatment on pain intensity assessed |
| 1. Exclusion criteria | Study participants <18 years old; study population <50% SCI; study <3 participants; acute setting; participants with musculoskeletal pain only; review articles; case reports/studies; study protocols; qualitative designs |

Appendix 1: Search Strategy

*Where possible, key words were searched under subject headings as well.
