# Supplementary material for: The CanPain SCI clinical practice guidelines for rehabilitation management of neuropathic pain after spinal cord injury: 2021 update
Source: Spinal Cord. 2022 Feb 5;60(6):548–66. doi: 10.1038/s41393-021-00744-z (PMC9209331; doi:10.1038/s41393-021-00744-z)
Supplement: Supplementary file 2 — Appendix 2 - GRADE scoring criteria [file 41393_2021_744_MOESM2_ESM.docx]

Appendix 2: GRADE Scoring Criteria

| **Study Design** | **Quality of Evidence** | **Lower If** | **Higher if** |
| --- | --- | --- | --- |
| RCT | High | ***Risk of Bias***  -1 serious  -2 very serious  ***Inconsistency***  -1 serious  -2 very serious  ***Indirectness***  -1 serious  -2 very serious  ***Imprecision***  -1 serious  -2 very serious  ***Publication Bias***  -1 likely  -2 very likely | ***Large Effect***  +1 large  +2 very large  ***Dose Response***  +1 evidence of a gradient  ***All Plausible Confounding***  +1 would reduce a demonstrated effect or  +1 would suggest a spurious effect when results show no effect |
| Quasi-RCT | Moderate |  |  |
| Observational Study | Low |  |  |
| Case Study | Very Low |  |  |

*Adapted from Guyatt G, et al. (2010).^6^*
