# Supplementary material for: The CanPain SCI clinical practice guidelines for rehabilitation management of neuropathic pain after spinal cord injury: 2021 update
Source: Spinal Cord. 2022 Feb 5;60(6):548–66. doi: 10.1038/s41393-021-00744-z (PMC9209331; doi:10.1038/s41393-021-00744-z)
Supplement: Supplementary file 3 — Appendix 3 - Visual representation of a clinical algorithm for the screening/diagnosis and treatment of neuropathic pain following SCI [file 41393_2021_744_MOESM3_ESM.pdf]

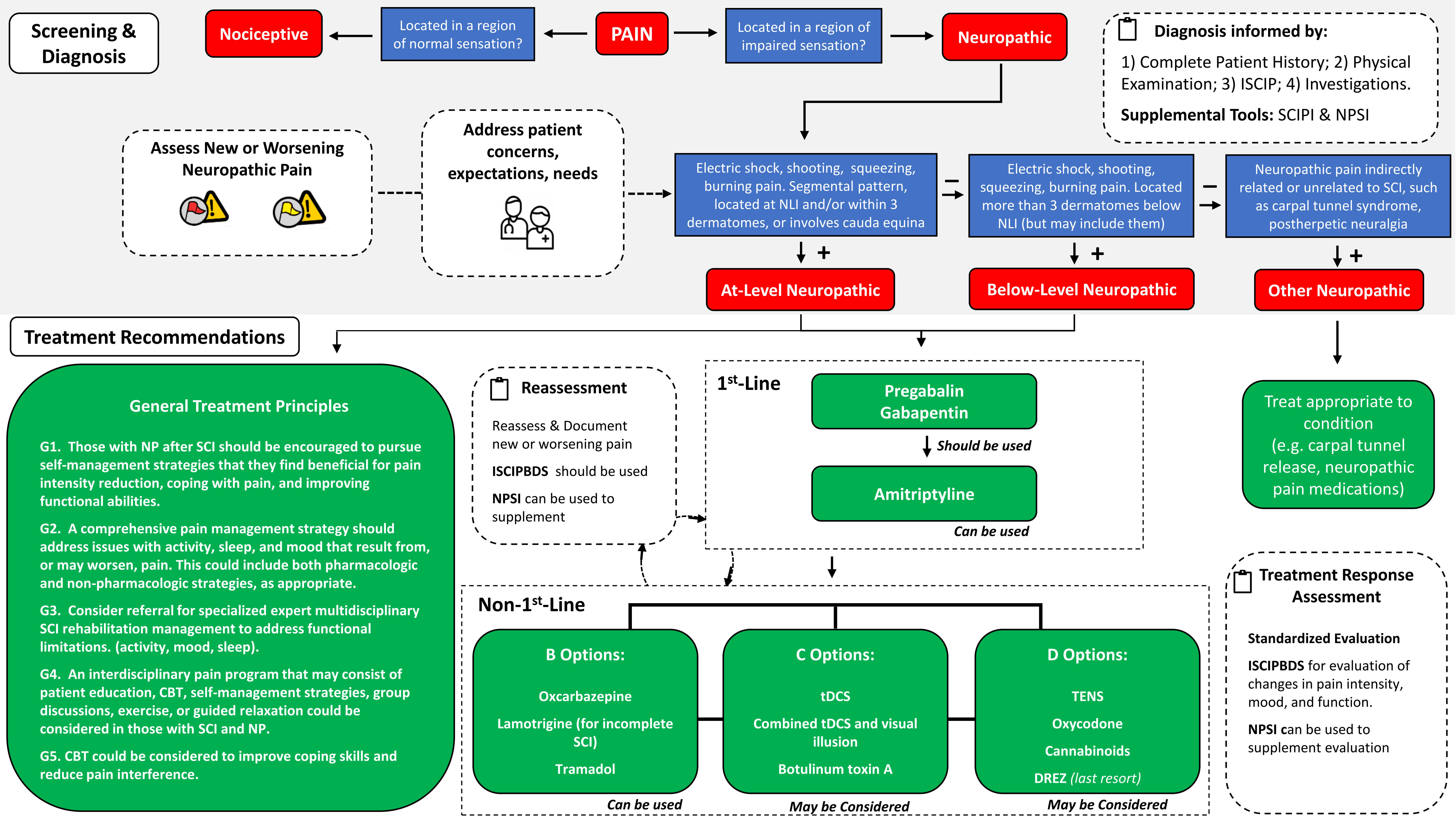
Screening & Diagnosis
Nociceptive
Located in a region of normal sensation?
PAIN
Located in a region of impaired sensation?
Neuropathic
Diagnosis informed by:
1) Complete Patient History; 2) Physical Examination; 3) ISCIP; 4) Investigations.
Supplemental Tools: SCIPI & NPSI
Assess New or Worsening Neuropathic Pain
Address patient concerns, expectations, needs
Electric shock, shooting, squeezing, burning pain. Segmental pattern, located at NLI and/or within 3 dermatomes, or involves cauda equina
Electric shock, shooting, squeezing, burning pain. Located more than 3 dermatomes below NLI (but may include them)
Neuropathic pain indirectly related or unrelated to SCI, such as carpal tunnel syndrome, postherpetic neuralgia
At-Level Neuropathic
Below-Level Neuropathic
Other Neuropathic
Treatment Recommendations
General Treatment Principles
G1. Those with NP after SCI should be encouraged to pursue self-management strategies that they find beneficial for pain intensity reduction, coping with pain, and improving functional abilities.
G2. A comprehensive pain management strategy should address issues with activity, sleep, and mood that result from, or may worsen, pain. This could include both pharmacologic and non-pharmacologic strategies, as appropriate.
G3. Consider referral for specialized expert multidisciplinary SCI rehabilitation management to address functional limitations. (activity, mood, sleep).
G4. An interdisciplinary pain program that may consist of patient education, CBT, self-management strategies, group discussions, exercise, or guided relaxation could be considered in those with SCI and NP.
G5. CBT could be considered to improve coping skills and reduce pain interference.
Reassessment
Reassess & Document new or worsening pain
ISCIPBDS should be used
NPSI can be used to supplement
1st-Line
Pregabalin
Gabapentin
Should be used
Amitriptyline
Can be used
Treat appropriate to condition (e.g. carpal tunnel release, neuropathic pain medications)
Non-1st-Line
B Options:
Oxcarbazepine
Lamotrigine (for incomplete SCI)
Tramadol
Can be used
C Options:
tDCS
Combined tDCS and visual illusion
Botulinum toxin A
May be Considered
D Options:
TENS
Oxycodone
Cannabinoids
DREZ (last resort)
May be Considered
Treatment Response Assessment
Standardized Evaluation
ISCIPBDS for evaluation of changes in pain intensity, mood, and function.
NPSI can be used to supplement evaluation
